# QUALITY OF LIFE AND ATTITUDES TOWARD AGING IN OLDER ADULTS DURING THE COVID-19 PANDEMIC

**DOI:** 10.1093/geroni/igac059.2005

**Published:** 2022-12-20

**Authors:** Morgan Inman, Maria MacLean, Olive Bryanton, William Montelpare, Jessica Strong

**Affiliations:** University of Prince Edward Island, Charlottetown, Prince Edward Island, Canada; University of Prince Edward Island, Charlottetown, Prince Edward Island, Canada; Prince Edward Island, Charlottetown, Prince Edward Island, Canada; University of Prince Edward Island, Charlottetown, Prince Edward Island, Canada; University of Prince Edward Island, Charlottetown, Prince Edward Island, Canada

## Abstract

Research has shown that positive or negative views of aging are associated with quality of life. Prior research has found that community dwelling older adults aged 65 and older with more negative views of aging have lower scores on quality of life scales, whereas those with higher views of aging have higher scores on quality of life scales. A group of 264 community dwelling older adults (Mean age = 72.4 years, 62-92 years old) living in Prince Edward Island, Canada, completed a survey measuring attitudes towards aging and quality of life during the COVID-19 pandemic. The sample consisted of a majority of retired (n=206) older adults, living in an urban area (n=151), and approximately 55% receiving a household income of $26,000 to $75,000 per year. Regression analysis found that attitudes towards aging significantly predicted quality of life (F(1,127)=24.9, p< 0.01), with positive attitudes predicting higher quality of life scores and negative attitudes predicting lower quality of life scores. The model showed that the predictor, attitudes towards aging, explained 16.5% of the variance in quality of life (B=-2.7, t=-4.9, p< 0.01). This suggests that attitudes towards aging play a role in predicting quality of life in older adults during the COVID-19 pandemic.

